# Unique expression patterns of the embryonal stem cell marker SOX2 and hormone receptors suggest the existence of a subpopulation of epithelial stem/progenitor cells in porcine and bovine endometrium

**DOI:** 10.1002/vms3.802

**Published:** 2022-05-13

**Authors:** Jiri Lenz, Petra Konecna, Frantisek Tichy, Dominika Machacova, Ludek Fiala, Pavel Hurnik, Michal Kyllar

**Affiliations:** ^1^ Department of Anatomy Histology and Embryology Faculty of Veterinary Medicine University of Veterinary Sciences Brno Brno Czech Republic; ^2^ Department of Pathology Znojmo Hospital Znojmo Czech Republic; ^3^ Cytohisto s.r.o. Břeclav Czech Republic; ^4^ Department of Sexology Psychiatric Clinic Faculty of Medicine Charles University Pilsen Pilsen Czech Republic; ^5^ Institute of Sexology First Faculty of Medicine Charles University Prague Prague Czech Republic; ^6^ CGB Laboratory Inc. Ostrava Czech Republic; ^7^ Department of Pathobiology Institute of Morphology University of Veterinary Medicine Vienna Vienna Austria

**Keywords:** endometrium, epithelial stem/progenitor cells, farm animals, hormone receptors, SOX2

## Abstract

**Background:**

There are currently insufficient data on the population of endometrial epithelial stem/progenitor cells in farm animals.

**Objectives:**

With the aim of identifying a potential population of epithelial stem/progenitor cells in the porcine and bovine endometrium, this study immunohistochemically examined the expression patterns of the oestrogen and progesterone receptors, as well as that of the embryonal stem cell marker SOX2.

**Methods:**

A total of 24 endometrial tissue samples obtained from cycling pigs (*n* = 12) and cows (*n* = 12) were included in our study. Each endometrium was divided into basal, middle and luminal portions. The percentage of marker‐positive cells and the intensity of the immunoreaction in each portion of the endometrium were determined.

**Results:**

Inverse expression patterns of SOX2 and progesterone receptors were found in both animal species throughout the oestrous cycle. Strong diffuse SOX2 expression was detected in the basal portions of the glands, while a significant decrease in positivity and a weak immunoreaction were found in the luminal two thirds of the glandular epithelium. Strong progesterone receptor expression was observed in at least 90% of glandular cells in the middle and luminal portions, whereas weak staining and significant decrease in positivity were detected in the basal portions of the glands. One oestrogen receptor expression pattern resembled that of progesterone receptors.

**Conclusion:**

The inverse expression patterns of SOX2 and hormone (especially progesterone) receptors suggest that endometrial epithelial stem/progenitor cells represent a subset of cells that reside in the basal portions of the endometrial glands in both the bovine and porcine endometrium.

## Graphical abstract

The inverse expression patterns of SOX2 and hormone (especially progesterone) receptors suggest that endometrial epithelial stem/progenitor cells represent a subset of cells that reside in the basal portions of the endometrial glands in both the bovine and porcine endometrium. SOX2 appears to be a promising marker for identifying the basal portions of the endometrial glands.

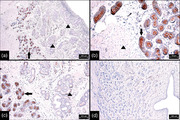

## 1 INTRODUCTION

Stem cells are undifferentiated cells characterized by the ability to self‐renew and differentiate into multiple cell types (Garget, 2004; He et al., 2009). Several types of stem cells are currently recognized. Totipotent stem cells, which have the highest differentiating potential, have the ability to develop into any cell within the whole organism. By contrast, pluripotent stem cells do not have the ability to form extraembryonic tissue. Multipotent stem cells have a limited differentiation capacity, but can produce cells of a specific cell lineage (e.g., mesenchymal stem cells in the endometrium can differentiate into several types of connective tissue cells). The remaining types are oligopotent stem cells and unipotent stem cells. Oligopotent stem cells retain a relatively broad differentiation capacity; for example, myeloid stem cells can produce erythrocytes, platelets and various white blood cells. By contrast, unipotent stem cells, such as muscle stem cells, have the most limited differentiation potential and can only produce one cell type. Progenitor cells are early progeny of stem cells, but unlike unipotent stem cells, they do not have the ability to self‐renew (Mitalipov & Wolf, 2009; Mutalibov & Totipotency, 2009; Trounson, 2006; Ulloa‐Montoya et al., 2005).

The enormous regenerative capacity of the human endometrium, together with its bilayer structure, in which the stratum functionalis is sloughed off during the menstrual cycle and is regenerated from the stratum basalis, has prompted researchers to investigate the existence of endometrial stem/progenitor cell population (Padykula, 1991). The endometrium of farm animals, including pigs and cows, undergoes specific morphological changes during the reproductive cycle, known as the oestrous cycle (Noseir, 2003; Soede et al., 2011). Despite many similarities, there are several substantial differences between the menstrual and oestrous cycles (i.e., the endometrium is reabsorbed during the oestrous cycle but shed during the menstrual cycle). In relation to our study, the differences that result from the microscopic structure of the endometrium itself are crucial. Unlike that in animals with oestrous cycles, the endometrium of humans undergoing menstrual cycles is divided into two structurally and functionally distinct layers: the stratum functionalis (upper layer) and the stratum basalis (lower layer). Both the morphological appearance and the thickness of the upper functional layer differ markedly during specific phases of the menstrual cycle. The lower basal layer abuts the myometrium, has a thickness of 0.5 to 1 mm and contains the bottoms of the uterine glands, capillaries and connective tissue cells, the proliferation of which leads to the restitution of the competent functional layer. Thus, it can be assumed that the persistent glands and connective tissue in the stratum basalis contain subpopulations of both epithelial progenitor cells and multipotent mesenchymal stem cells (Ferenczy, 1976; Salamonsen, 2003; Spencer et al., 2005). However, there are currently very few publications describing the identification of epithelial progenitor cells and their markers in the endometrium of both humans and animals. In particular, the localization of endometrial epithelial stem/progenitor cells in animal species undergoing the oestrous cycle remains unknown.

With the aim of identifying a potential population of endometrial epithelial stem/progenitor cells in the bovine and porcine endometrium, this study examined the expression patterns of the oestrogen receptor alpha (ER) and progesterone receptor (PR), as well as that of the embryonal stem cell marker SOX2 using immunohistochemical staining methods. Regarding the association between hormone receptors and epithelial progenitor cells in the endometrium, a lower content of hormone receptors is thought to indicate a less differentiated cell phenotype, which is a typical feature of progenitor cells (Valentijn et al., 2013). We also examined the expression of the embryonal stem cell marker SOX2, a member of the sex determining region Y (SRY)‐related HMG box family of transcriptional factors that plays a key role in mammalian development. SOX2 is essential for maintaining pluripotency in undifferentiated embryonic and neural stem cells and is considered a promising marker in the field of induced pluripotency (Bunina et al., 2020; Holmes et al., 2020).

## 2 MATERIALS AND METHODS

### 2.1 Animals and tissue specimens

A total of 24 resection specimens consisting of uterine cervix, uterine corpus, uterine horns, fallopian tubes and ovaries were obtained from healthy cycling pigs (*n* = 12) and cows (*n* = 12) slaughtered in an animal abattoir. Tissue fragments measuring approximately 2 × 1,5 × 1 cm was dissected from the middle parts of both uterine horns of each sample. In addition, both ovaries were separated from the resection specimens and cut in a longitudinal plane into two equal parts. The obtained tissue fragments were then fixed in 10% neutral buffered formalin for 36 h (1.5 days). Further tissue processing was performed in a standard manner. In brief, the samples were dehydrated by immersion in ethanol solutions of increasing concentrations, and then cleared with xylene and wax infiltration. In the final step, thin tissue sections (3–4 μm) were stained with haematoxylin‐eosin.

The phase of the oestrous cycle was determined by gross examination of both ovaries according to the criteria described by Ireland et al. (Ireland et al., 1980) and confirmed microscopically by evaluating the histological appearance of endometrial tissue and folliculogenesis and/or luteogenesis of both ovaries (Ginther et al., 1989). The following morphological criteria were used to evaluate the endometrium and ovaries: proestrus (elongation of endometrial glands, onset of stromal oedema, dilation of blood vessels, increasing number of fibroblasts in the endometrial mucosa and maturation of ovarian follicles); oestrus (onset of secretory changes in glandular cells, mild stromal oedema and congestion, rupture of Graafian follicles with the presence of a fibrin core); early metestrus (increasing stromal oedema, congestion and secretory glandular changes together with the onset of corpus luteum formation); mid and late metestrus (highly developed glandular secretory changes along with stromal oedema and congestion and a well‐formed corpus luteum); dioestrus (regression of both the uterine mucosa and the corpus luteum). Proestrus and oestrus (which is the period of ovulation) correspond to the follicular phase, while metestrus and dioestrus correspond to the luteal phase of the oestrous cycle.

For immunohistochemical analyses, the endometrium was divided into basal, middle and luminal portions. While the thicknesses of the middle and luminal portions varied depending on the presence of mucosal folds, that of the basal portion of the endometrial mucosa was limited to 0.5 mm (similar to the stratum basalis in the human endometrium). As the endometrium of farm animals is not organized into functionalis and basalis layers, we replace the term basalis glandular epithelium with a descriptive term: the basal portion of the endometrial glands.

### 2.2 Immunohistochemistry

Three antibodies were used in immunohistochemical assay, namely ER (clone 1D5, dilution 1:200, DakoCytomation GmBH, Hamburg, Germany), PR (clone SP2, dilution 1:200, DCS Innovative Diagnostik‐Systeme, Hamburg, Germany) and SOX2 (rabbit polyclonal, dilution 1:75, Antibodies‐online GmBH, Aachen, Germany, catalogue number ABIN2777428, immunogen is a synthetic peptide directed towards the N terminal region of human SOX2, antigen size 317 AA, molecular weight 34 kDa). Immunohistochemical staining was performed using the automated immunostainer BenchMark Ultra (Ventana Medical System Inc., Oro Valley, Arizona, USA) and the ultraView Universal DAB Detection Kit (Ventana Medical System Inc.). The porcine and bovine endometrial tissue samples were incubated with the anti‐ER, anti‐PR and anti‐SOX2 antibodies for 20, 32 and 16 min, respectively. Prior to immunohistochemical staining, heat‐induced antigen retrieval was performed in Tris‐ethylenediaminetetraacetic acid (EDTA)‐based buffer (pH 8.4) for 20 min (ER) or 64 min (PR and SOX2) at 95°C (Ventana Medical System Inc.).

External positive controls were used for all three antibodies (human endometrium for ER and PR, and human spleen for SOX2). Negative controls were prepared by incubating samples with diluted rabbit serum (dilution 1:75). The porcine and bovine myometrium served as a positive internal control for hormone receptors. Evaluation of immunohistochemical assays was performed using the following type of microscope and camera: Olympus BX53 microscope and Promican 3‐3CC camera (Tokyo, Japan).

### 2.3 Evaluation of immunostaining

For ER and PR, nuclear staining in the endometrium and myometrium was considered positive. For SOX2, cytoplasmic and nuclear staining in endometrial stroma and cytoplasmic staining in endometrial glands (Perry et al., 2013) were considered positive. ER and PR expression was evaluated in endometrial glandular cells only, while SOX2 positivity was assessed in both endometrial glands and stroma. The percentages of marker‐positive cells were evaluated using a light microscope at ×200 magnification. At least five foci of each portion (basal, middle and luminal) of the porcine and bovine endometrial sections were analyzed, and the percentage of marker‐positive cells of the covered area was determined. The intensity of the immunoreaction was determined as weak, moderate or strong (standard practice in surgical pathology). The assessment of immunohistochemical staining was evaluated independently by two histopathologists (Jiri Lenz and Frantisek Tichy). Discrepancies were resolved by a consensus.

### 2.4 Statistical analysis

Using statistical analysis, differences in the percentages of marker‐positive cells between the basal portions and the luminal two thirds (i.e., middle and luminal portions) of the endometrial glands were determined in each endometrial sample. Differences were compared using the McNemar's test for paired nominal data. *α* = 0.05 was used as the level of statistical significance in all analyses.

## 3 RESULTS

### 3.1 Microscopic findings of porcine and bovine endometrium and ovaries

**FIGURE 1:**
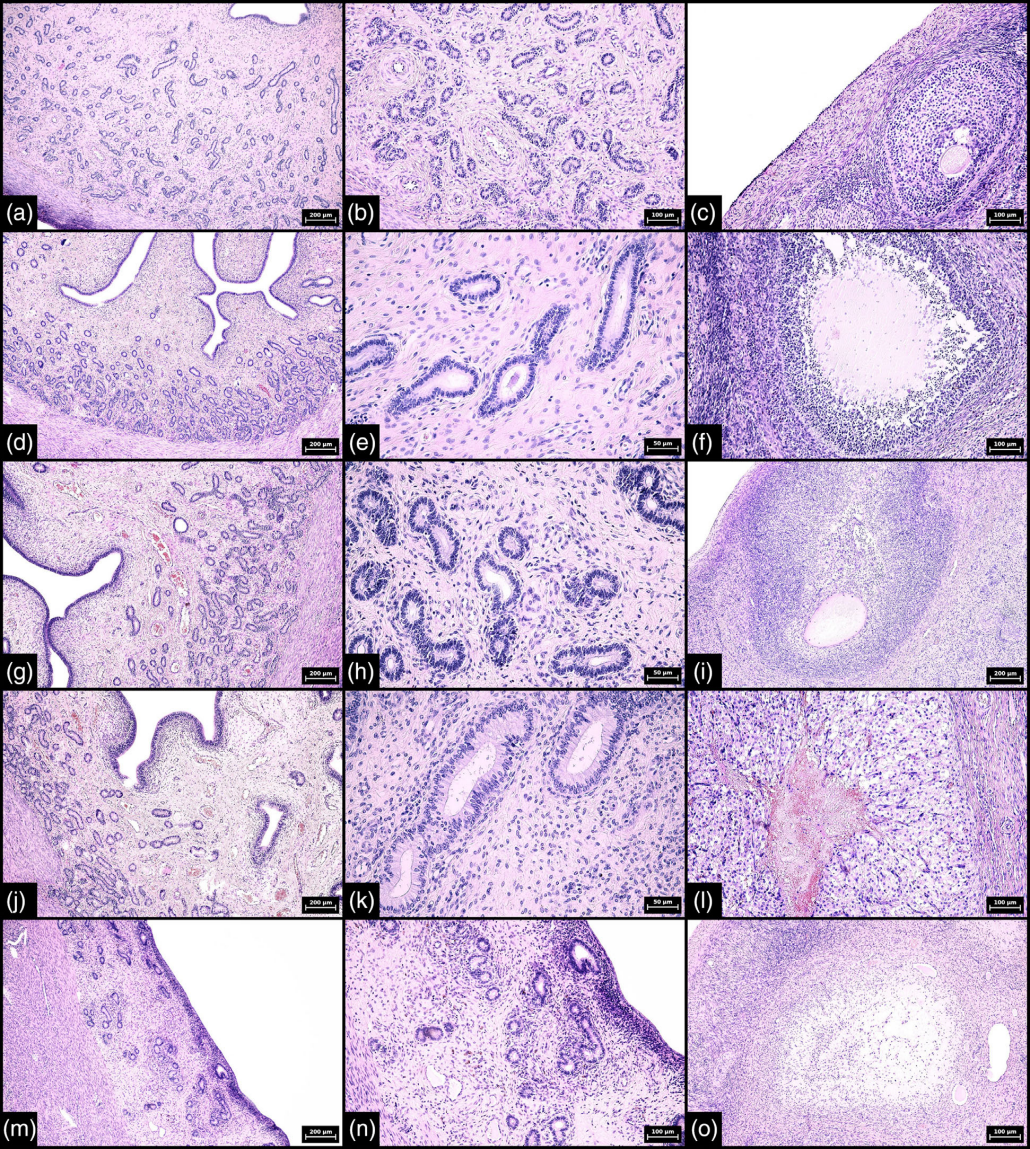
Morphological features of porcine endometrium and corresponding ovary in proestrus, oestrus, metestrus and dioestrus (haematoxylin‐eosin staining). (a and b) Proestrus characterized by elongation of endometrial glands, onset of stromal oedema, dilation of blood vessels and increasing number of fibroblasts in the endometrial mucosa (100× magnification, scale bar 200 μm [a], 200× magnification, scale bar 100 μm [b]). (c) Ovary with maturing follicle; proestrus (200× magnification, scale bar 100 μm). (d and e) Oestrus characterized by mild stromal oedema, congestion and the onset of secretory changes in the endometrial glands (100× magnification, scale bar 200 μm [d], 400× magnification, scale bar 50 μm [e]). (f) Ovary with a ruptured Graafian follicle with a centrally located fibrin core; oestrus (200× magnification, scale bar 100 μm). (g and h) Early metestrus characterized by increasing stromal oedema, congestion and secretory glandular changes (100× magnification, scale bar 200 μm [g], 400× magnification, scale bar 50 μm [h]). (i) Ovary with a ruptured Graafian follicle showing the onset of corpus luteum formation; early metestrus (100× magnification, scale bar 200 μm). (j and k) Mid to late metestrus characterized by marked congestion, stromal oedema and secretory glandular changes (100× magnification, scale bar 200 μm [j], 400× magnification, scale bar 50 μm [k]). (l) Ovary with a well‐formed corpus luteum; mid to late metestrus (200× magnification, scale bar 100 μm). (m and n) Dioestrus characterized by regression of the uterine mucosa (100× magnification, scale bar 200 μm [m], 200× magnification, scale bar 100 μm [n]). (o) Ovary with regression of the corpus luteum; dioestrus (200× magnification, scale bar 100 μm). The numerical aperture (NA) of the objective lens: 0.25, 0.40 and 0.65 for 100×, 200× and 400× magnification, respectively

In all cases, the morphological appearance of the endometrium was completely synchronous with the folliculogenesis and luteogenesis of both ovaries. Regarding porcine tissue samples, two cases were classified as proestrus (corresponding to days 17–21 of the cycle) (Figure 1a–c), two cases as oestrus (corresponding to days 1–2 of the cycle) (Figure 1d–f), four cases as early metestrus (corresponding to days 3–4 of the cycle) (Figure 1g–i), two cases as mid/late metestrus (corresponding to days 5–8 of the cycle) (Figure 1j–l) and two cases as dioestrus (corresponding to days 9–17 of the cycle) (Figure 1m–o). For bovine tissue samples, three cases were classified as proestrus, two cases as oestrus, three cases as early metestrus, two cases as mid/late metestrus and two cases as dioestrus.

### 3.2 Analysis of SOX2 expression in porcine and bovine endometrium

**FIGURE 2:**
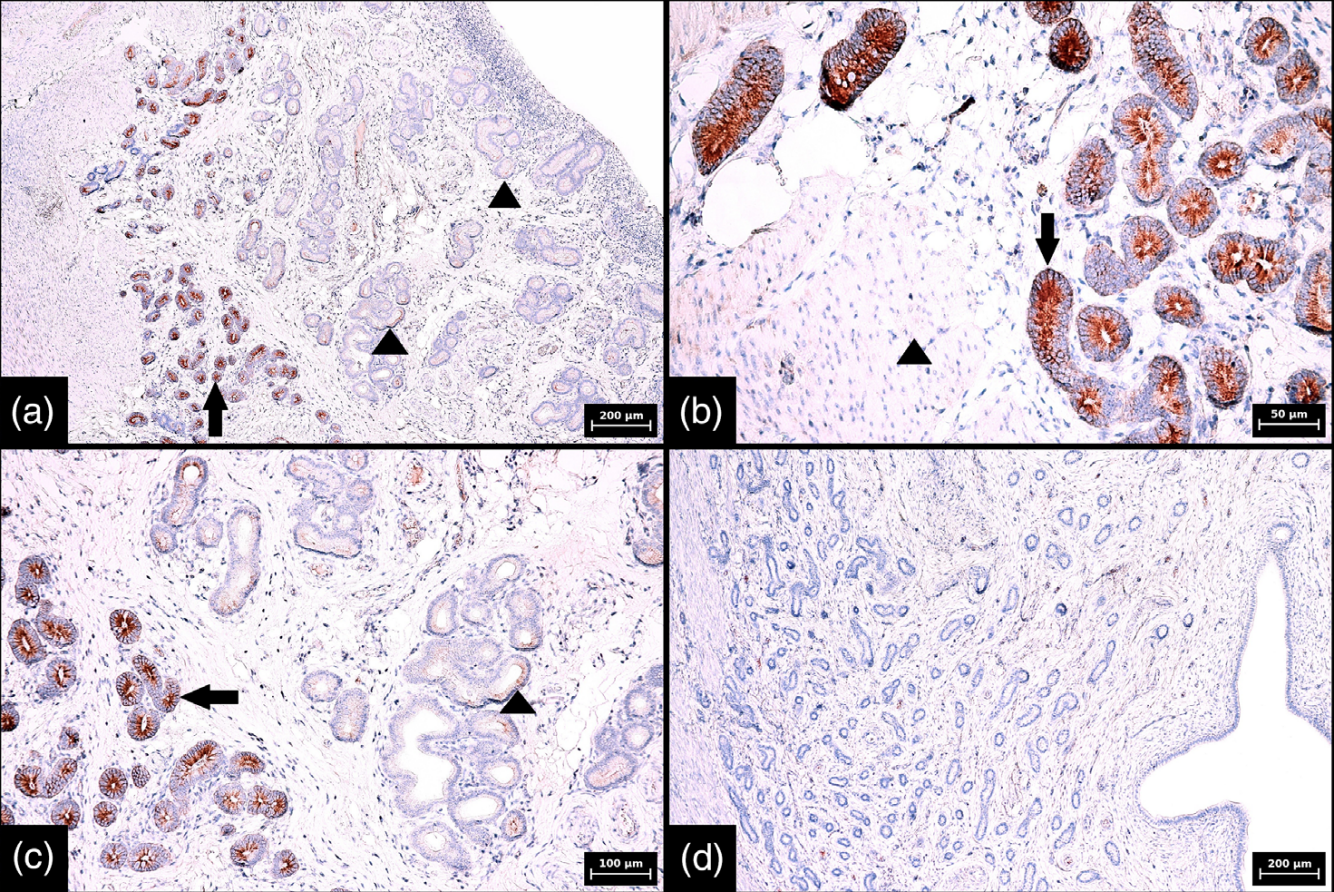
Immunohistochemical expression of SOX2 in glands in porcine endometrium during metestrus and negative control. (a) Low magnification showing a difference in SOX2 expression between individual portions of endometrial glands, namely strong diffuse expression in basal portions (arrow) and weak and sporadic expression in middle and luminal portions of glands (arrowheads) (100× magnification, scale bar 200 μm). (b) Interface between strongly stained basal portions of endometrial glands (arrow) and negative leiomyocytes of the myometrium (arrowhead) (400× magnification, scale bar 50 μm). (c) Interface between strongly and diffusely stained basal portions (arrow) and sporadically and weakly stained middle portions (arrowhead) of endometrial glands (200× magnification, scale bar 100 μm). (d) Negative control in which the SOX‐2 antibody was replaced by diluted rabbit serum; completely negative endometrial glands and stroma are apparent (100× magnification, scale bar 200 μm). Numerical aperture (NA) of the objective lens: 0.25, 0.40 and 0.65 for 100×, 200× and 400× magnification, respectively

**FIGURE 3:**
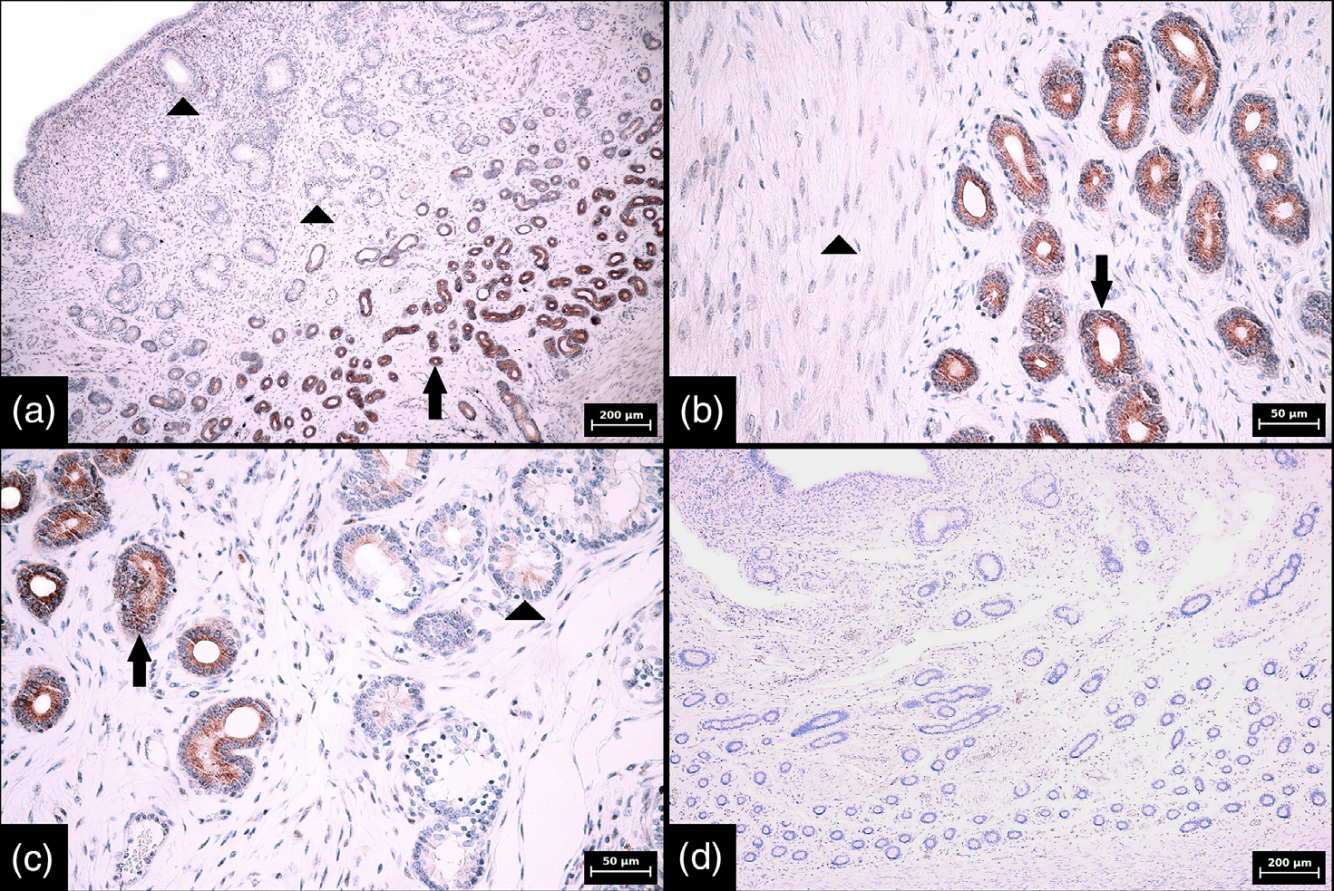
Immunohistochemical expression of SOX2 in glands in bovine endometrium during oestrus and negative control. (a) Low magnification showing a difference in SOX2 expression between the individual portions of endometrial glands, namely strong and diffuse expression in basal portions (arrow) and weak and sporadic expression in middle and luminal portions of glands (arrowheads) (100× magnification, scale bar 200 μm). (b) Interface between strongly stained basal portions of endometrial glands (arrow) and negative leiomyocytes (arrowhead) of the myometrium (400× magnification, scale bar 50 μm). (c) Interface between strongly and diffusely stained basal portions (arrow) and sporadically and weakly stained middle portions of endometrial glands (arrowhead) (400× magnification, scale bar 50 μm). (d) Negative control in which the SOX‐2 antibody was replaced by diluted rabbit serum; completely negative endometrial glands and stroma are apparent (100× magnification, scale bar 200 μm). Numerical aperture (NA) of the objective lens: 0.25 for 100× magnification and 0.65 for 400× magnification

**FIGURE 4:**
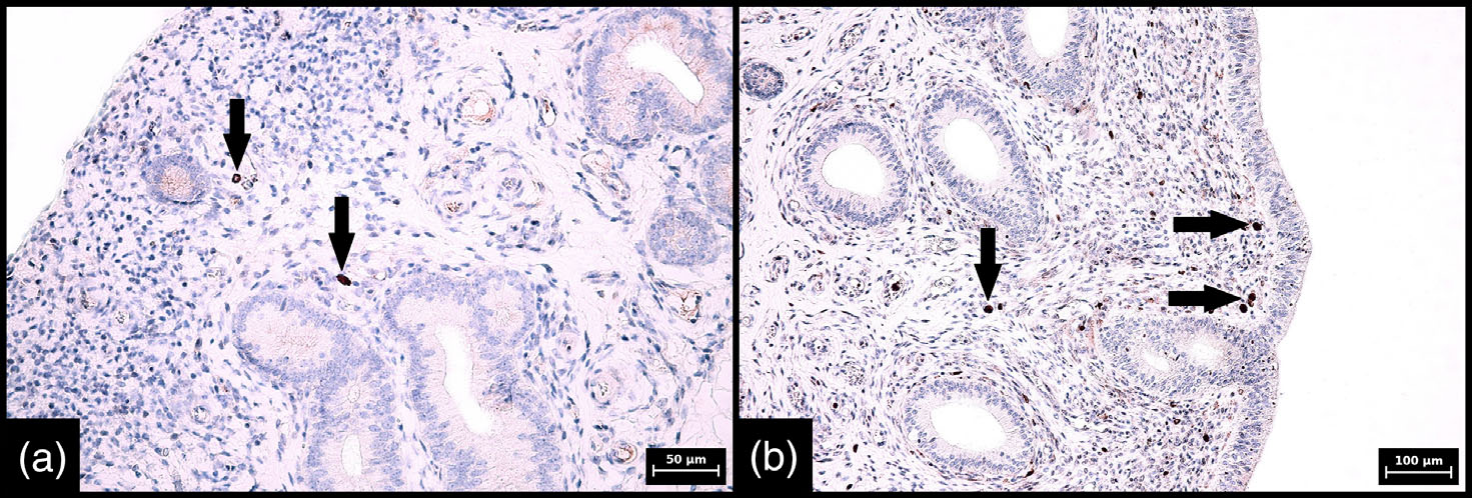
Immunohistochemical expression of SOX‐2 in stromal cells in porcine and bovine endometrium. Sporadic and strong SOX‐2 expression in endometrial stromal cells (arrows) in porcine (a) and bovine (b) endometrium; most SOX2‐positive stromal cells are located in the superficial endometrium (400× magnification, scale bar 50 μm [a], 200× magnification, scale bar 100 μm [b]). Numerical aperture (NA) of the objective lens: 0.40 for 200× magnifications and 0.65 for 400× magnification

Identical SOX2 expression patterns were found in porcine and bovine endometrium regardless of the phase of the oestrous cycle (Tables 1 and 2). In both animal species, SOX2 expression in glands was detected mainly in the basal portion of the endometrial tissue and was characterized by strong and diffuse cytoplasmic positivity in all glandular cells. By contrast, in all samples examined, less than or equal to 20% of the cells in the glands in the middle and luminal portions were focally positive and the reaction was weak (*p* < 0.001) (Figures 2a–c and 3a–c). Negative controls are also illustrated (Figures 2d and 3d). In the endometrial stroma, strong cytoplasmic and nuclear positivity was found in less than 1% of cells. Distribution of these marker‐positive stromal cells was random, and clustering was not apparent. Most SOX2‐positive stromal cells were located just below the surface epithelium (Figure 4).

**TABLE 1:** Cycling status of the study group and immunohistochemical analyses of SOX2 and oestrogen and progesterone receptors in basal portions of endometrial glands in porcine and bovine endometrium

Porcine endometrium					Bovine endometrium			
Case	Cycling status	SOX2^a^(II)	ER^a^(II) EREP	PR^a^(II)	Cycling status	SOX2^a^(II)	ER^a^(II) EREP	PR^a^(II)
1	Proestrus	100(s)	70(w) 1	50(w)	Proestrus	100(s)	>90(s) 1	25(w)
2	Proestrus	100(s)	80(w) 2	60(w)	Proestrus	100(s)	>90(s) 1	20(w)
3	Oestrus	100(s)	80(w) 2	65(w)	Proestrus	100(s)	>95(s) 3	10(w)
4	Oestrus	100(s)	80(w) 2	55(w)	Oestrus	100(s)	>90(s) 1	25(w)
5	Early metestrus	100(s)	80(w) 2	50(w)	Oestrus	100(s)	60(w) 2	20(w)
6	Early metestrus	100(s)	80(w) 2	65(w)	Early metestrus	100(s)	60(w) 2	20(w)
7	Early metestrus	100(s)	80(w) 2	50(w)	Early metestrus	100(s)	60(w) 2	30(w)
8	Early metestrus	100(s)	80(w) 2	50(w)	Early metestrus	100(s)	>95(s) 3	15(w)
9	Mid/late metestrus	100(s)	80(w) 2	50(w)	Mid/late metestrus	100(s)	60(w) 2	10(w)
10	Mid/late metestrus	100(s)	80(w) 2	50(w)	Mid/late metestrus	100(s)	>95(s) 3	30(w)
11	Dioestrus	100(s)	75(w) 1	50(w)	Dioestrus	100(s)	>95(s) 3	15(w)
12	Dioestrus	100(s)	80(w) 2	65(w)	Dioestrus	100(s)	>95(s) 3	25(w)

Abbreviations: ER, oestrogen receptor; EREP, oestrogen receptor expression pattern; II, immunostaining intensity; m, moderate; PR, progesterone receptor; s, strong; w, weak.

^a^ Percentage (%) of marker positive endometrial glandular cells.

**TABLE 2:** Cycling status of the study group and immunohistochemical analyses of SOX2 and oestrogen and progesterone receptors in the luminal two thirds of endometrial glands in porcine and bovine endometrium

Porcine endometrium					Bovine endometrium			
Case	Cycling status	SOX2^a^(II)	ER^a^(II) EREP	PR^a^(II)	Cycling status	SOX2^a^(II)	ER^a^(II) EREP	PR^a^(II)
1	Proestrus	15(w)	90(w) 1	95(s)	Proestrus	10(w)	>90(s/m) 1	>70(s)
2	Proestrus	10(w)	90(w) 2	90(s)	Proestrus	15(w)	>90(s/m) 1	>80(s)
3	Oestrus	20(w)	>90(m/w) 2	95(s)	Proestrus	15(w)	>95(s) 3	>75(s)
4	Oestrus	15(w)	>90(m/w) 2	90(s)	Oestrus	5(w)	>90(s/m) 1	>80(s)
5	Early metestrus	15(w)	>90(m/w) 2	90(s)	Oestrus	5(w)	>90(m/w) 2	>75(s)
6	Early metestrus	10(w)	>90(m/w) 2	90(s)	Early metestrus	10(w)	>90(m/w) 2	>85(s)
7	Early metestrus	5(w)	>90(m/w) 2	95(s)	Early metestrus	15(w)	>90(m/w) 2	>60(s)
8	Early metestrus	10(w)	95(m/w) 2	95(s)	Early metestrus	20(w)	>95(s) 3	>70(s)
9	Mid/late metestrus	15(w)	90(m/w) 2	95(s)	Mid/late metestrus	5(w)	>90(m/w) 2	>75(s)
10	Mid/late metestrus	15(w)	>90(m/w) 2	95(s)	Mid/late metestrus	10(w)	>95(s) 3	>70(s)
11	Dioestrus	10(w)	90(w) 1	90(s)	Dioestrus	15(w)	>95(s) 3	>80(m)
12	Dioestrus	20(w)	>90(m/w) 2	95(s)	Dioestrus	15(w)	>95(s) 3	>75(m)

Abbreviations: ER, oestrogen receptor; EREP, oestrogen receptor expression pattern; II, immunostaining intensity; m, moderate; m/w, moderate reaction in the middle portions and weak reaction in the luminal glandular portions; PR, progesterone receptor; s, strong; s/m, strong reaction in the middle portions and moderate reaction in the luminal glandular portions; w, weak.

^a^ Percentage (%) of marker positive endometrial glandular cells.

### 3.3 Analysis of PR expression in porcine and bovine endometrium

**FIGURE 5:**
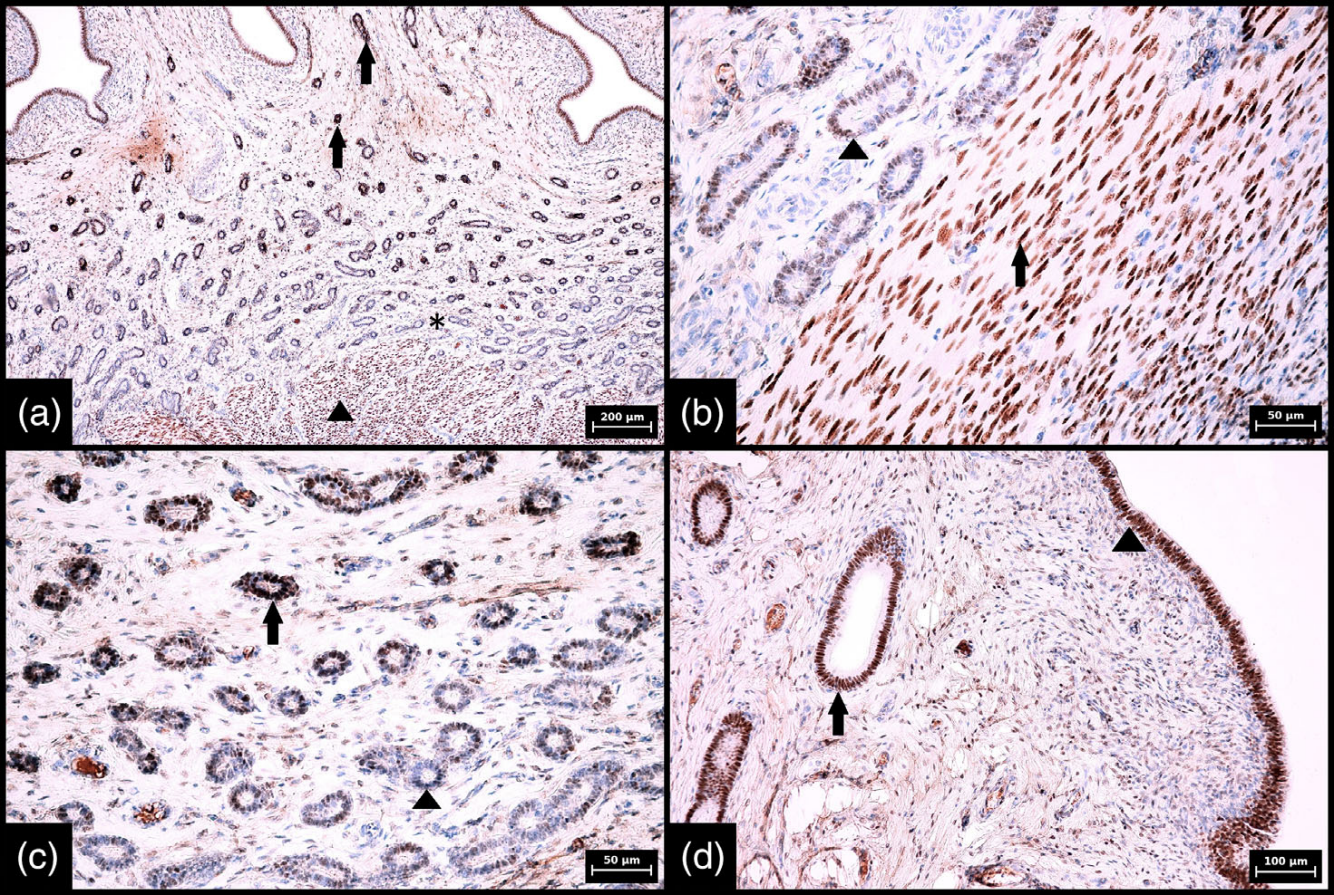
Immunohistochemical expression of progesterone receptors in porcine endometrium and myometrium during proestrus. (a) Low magnification showing a difference in the expression of progesterone receptors between individual portions of endometrial glands and myometrium, namely strong and almost diffuse expression in middle and luminal portions of glands (arrows) and myometrium (arrowhead) and weak and focal expression in basal portions of endometrial glands (*) (100× magnification, scale bar 200 μm). (b) Interface between strongly stained leiomyocytes (arrow) and weakly and focally stained basal portions of endometrial glands (arrowhead) (400× magnification, scale bar 50 μm). (c) Interface between strongly and almost diffusely stained middle portions (arrow) and focally and weakly stained basal portions of endometrial glands (arrowhead) (400× magnification, scale bar 50 μm). (d) Strongly and diffusely stained luminal portions of endometrial glands (arrow) and the surface epithelium (arrowhead) (200× magnification, scale bar 100 μm). Numerical aperture (NA) of the objective lens: 0.25, 0.40 and 0.65 for 100×, 200× and 400× magnification, respectively

Immunohistochemical staining of PR was similar in both porcine and bovine endometrium regardless of the phase of the oestrous cycle (Tables 1 and 2). Expression of PR varied significantly depending on the portion of the endometrium analyzed. In pigs, weak nuclear PR expression was observed in the basal portion of the endometrium (in 50%–70% of glandular cells). By contrast, in the middle and luminal portions, strong expression was found in more than 90% and 95% of glandular cells, respectively, with the highest values being reached in the surface epithelium (almost 100%) (Figure 5a–d). Compared with the basal portion, differences reached statistical significance (*p* < 0.001).

**FIGURE 6:**
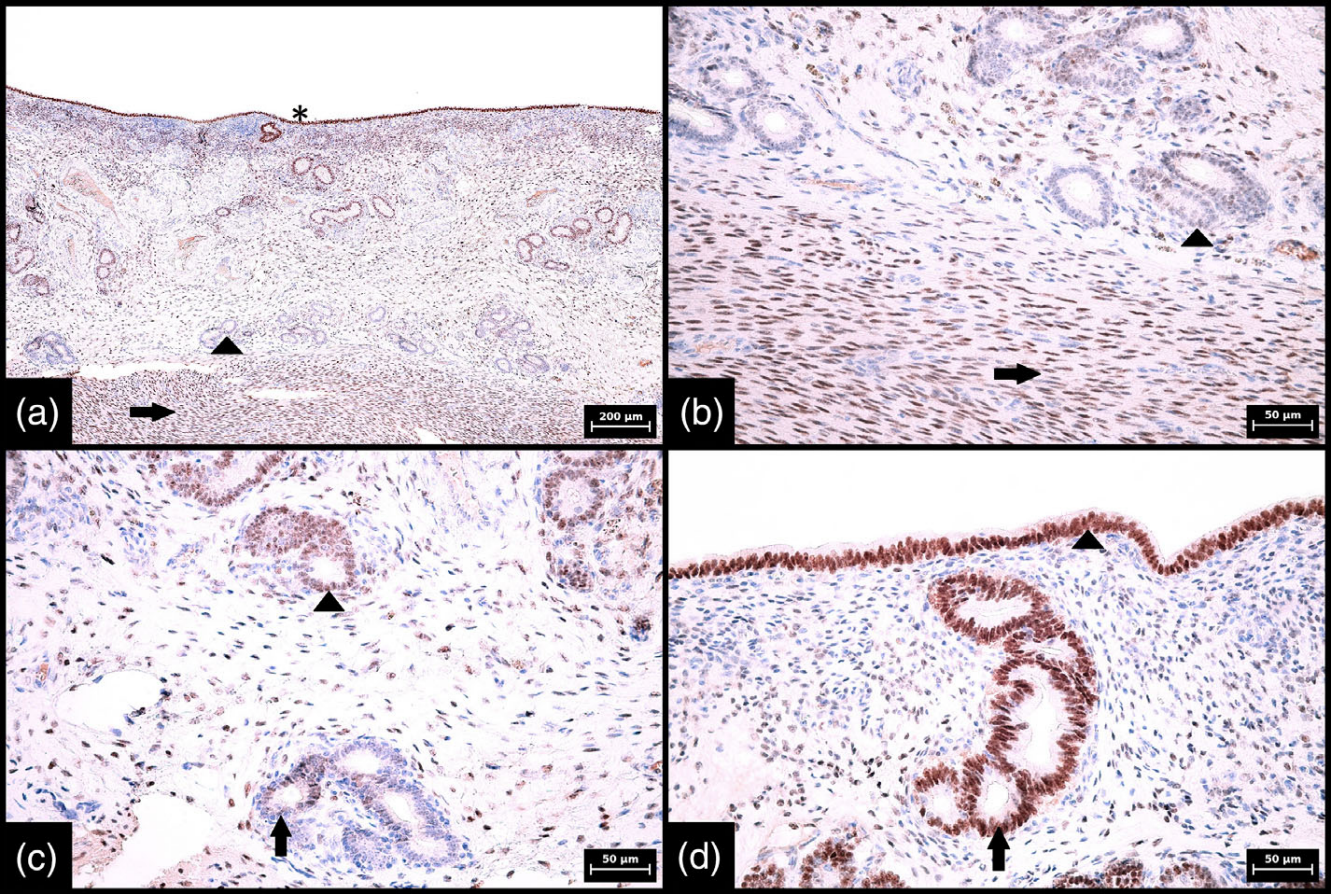
Immunohistochemical expression of progesterone receptors in bovine endometrium and myometrium during dioestrus. (a) Low magnification showing a difference in expression of progesterone receptors between individual portions of endometrial glands and myometrium, namely strong and diffuse expression in myometrium (arrow) and a gradual increase in intensity of the reaction and positivity of progesterone receptors from basal portions of glands (arrowhead) to the surface epithelium (*) (100× magnification, scale bar 200 μm). (b) Interface between strongly stained leiomyocytes (arrow) and weakly and focally stained basal portions of endometrial glands (arrowhead) (400× magnification, scale bar 50 μm). (c) Interface between weakly and sporadically stained basal portions (arrow) and multifocally and moderately stained middle portions of endometrial glands (arrowhead) (400× magnification, scale bar 50 μm). (d) Strongly and diffusely stained luminal portions of endometrial glands (arrow) and the surface epithelium (arrowhead) (400× magnification, scale bar 50 μm). Numerical aperture (NA) of the objective lens: 0.25 for 100× magnifications and 0.65 for 400× magnification

Only minor differences were observed in bovine endometrial samples. A gradual increase in the intensity of the reaction and the positivity of PR from the basal portion to the surface epithelium was evident (compared to a more abrupt transition between the basal and middle portions in pigs). The average percentages of PR‐positive cells in the bovine samples were 17% (range 5%–30%) in the basal portion, 70% (range 60%–80%) in the middle portion and >95% in the luminal portion. Regarding the intensity of the reaction, the only difference (compared with pigs) was the moderate expression in the middle portion of the endometrium observed in two cases (Figure 6a–d).

### 3.4 Analysis of ER expression in bovine endometrium

**FIGURE 7:**
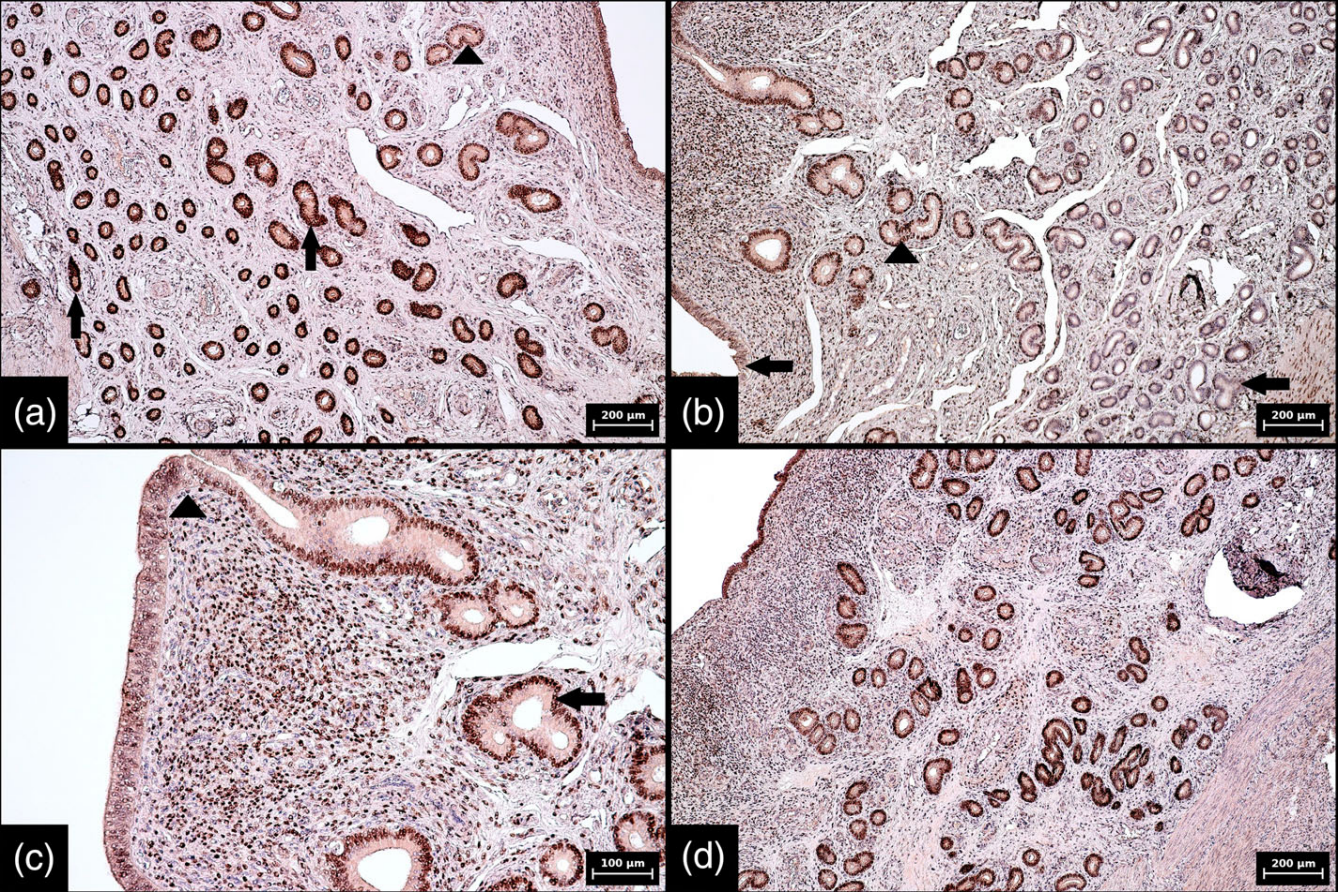
Immunohistochemical expression of oestrogen receptors in bovine endometrium during proestrus and metestrus. (a) The first expression pattern characterized by strong (basal and middle portions [arrows]) and moderate (luminal portions of glands [arrowhead]) positivity in more than 90% of glandular cells; proestrus (100× magnification, scale bar 200 μm). (b) The second expression pattern characterized by weak (basal and luminal portions [arrows]) and moderate (middle portions [arrowhead]) positivity and an increase in expression from 60% (basal portions) to more than 90% of glandular cells (remaining portions of glands); metestrus (100× magnification, scale bar 200 μm). (c) The second expression pattern at higher magnification showing moderate (middle portions [arrow]) and weak (luminal portions of glands [arrowhead]) positivity in more than 90% of glandular cells; metestrus (200× magnification, scale bar 100 μm). (d) The third expression pattern characterized by strong and diffuse expression across endometrial glands, including surface epithelium; proestrus (100× magnification, scale bar 200 μm). Numerical aperture (NA) of the objective lens: 0.25 for 100× magnification and 0.40 for 200× magnification

Three different ER expression patterns were found in bovine endometrial samples. The first pattern was characterized by virtually diffuse nuclear positivity in more than 90% of glandular cells across the endometrium (Tables 1 and 2). The reaction was strong in the basal and middle portions and moderate in the luminal portion of the endometrium (found in two cases classified as proestrus and one case classified as oestrus) (Figure 7a). The second expression pattern was characterized by weak expression in approximately 60% of the cells in the basal portion, moderate expression in more than 95% of cells in the middle portion and weak expression in more than 90% of the surface epithelium (*p* < 0.001) (found in one case classified as oestrus, three cases classified as metestrus) (Figure 7b,c). Significant differences in the percentage of marker‐positive cells between the basal portions and luminal two thirds of the endometrial glands indicate that the second ER expression pattern was similar to that of the PR. The third expression pattern was characterized by strong and diffuse ER expression throughout the endometrial mucosa (found in five cases, one classified as proestrus, two cases classified as metestrus and two cases classified as dioestrus) (Figure 7d).

### 3.5 Analysis of ER expression in porcine endometrium

**FIGURE 8:**
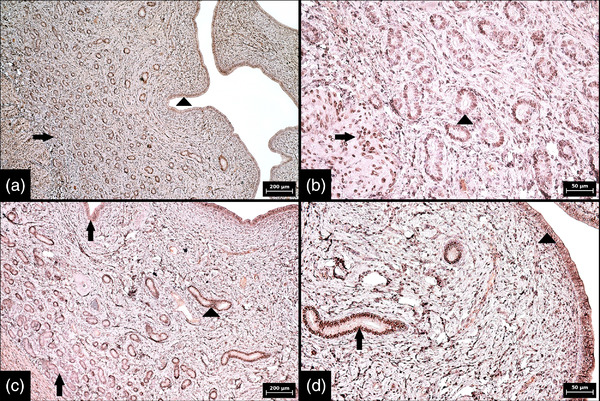
Immunohistochemical expression of oestrogen receptors in porcine endometrium during proestrus and metestrus. (a) The first expression pattern characterized by weak positivity and a gradual but small increase in expression from 70%–80% of glandular cells (basal portion [arrow]) to approximately 90% of cells (surface epithelium [arrowhead]); proestrus (100× magnification, scale bar 200 μm). (b) The first expression pattern showing the interface between moderately stained leiomyocytes (arrow) and weakly stained basal portions of endometrial glands (arrowhead); proestrus (400× magnification, scale bar 50 μm). (c) The second expression pattern characterized by weak (basal and luminal portions [arrows]) and moderate (middle portions [arrowhead]) positivity and a slight increase in expression from 70% (basal portions) to more than 90% of glandular cells (remaining portions of glands); metestrus (100× magnification, scale bar 200 μm). (d) The second expression pattern at higher magnification showing moderate (middle portions of glands [arrow]) and weak (surface epithelium [arrowhead]) positivity in more than 90% of cells; metestrus (400× magnification, scale bar 50 μm). Numerical aperture (NA) of the objective lens: 0.25 for 100× magnifications and 0.65 for 400× magnification

ER staining revealed two different expression patterns in porcine endometrial samples (Tables 1 and 2). A gradual slight increase in expression from 70%–80% of glandular cells in the basal portion to approximately 90% of the surface epithelium was observed in two cases classified as proestrus and one case classified as dioestrus. This expression pattern was characterized by a weak intensity of immunoreaction (Figure 8a,b). The second ER expression pattern was characterized by weak staining in approximately 80% of cells in the basal portion, moderate staining in the middle portion and weak staining in the luminal portion in more than 90% of cells (found in nine cases, two classified as oestrus, six as metestrus and one as dioestrus) (Figure 8c,d). Differences in the percentage of marker‐positive cells between the basal portions and luminal two thirds of the endometrial glands indicate that the second ER expression pattern was similar to that of the PR.

## 4 DISCUSSION

There are two limiting factors in endometrial stem cell research: non‐specific histological features and the absence of reliable (specific) markers for this cell population. Consequently, endometrial stem cell research in farm animals is scarce. To date, most studies have been performed on humans and have focused on endometrial mesenchymal stem cells. By contrast, the endometrial epithelial stem/progenitor cell population has not yet been characterized in farm animals undergoing the oestrous cycle. The results presented here suggest the existence of a subpopulation of epithelial stem/progenitor cells in the porcine and bovine endometrium.

Only a few publications have analyzed stem cell markers in the porcine and bovine endometrium using immunohistochemical staining methods. Our current study focused on the pluripotency marker SOX2, which is crucial for the survival and self‐renewal of undifferentiated embryonic stem cells. A search of the literature identified three studies reporting SOX2 positivity in the bovine endometrium (Cabezas et al., 2014; Lara et al., 2017; Łupicka et al., 2015) but no studies reporting SOX2 immunohistochemical expression in pigs. The first bovine finding was published by Cabezas et al. in 2014 (Cabezas et al., 2014). The animals included in this study were divided into two groups based on the stage of the oestrous cycle. Cases in the first group were classified as early luteal phase (days 1–5 of the cycle), while the second group included cases from the late luteal phase (days 13–18 of the cycle). Weak SOX2 positivity in the endometrium during the early luteal phase was described but not illustrated, while expression in the endometrium during the late luteal phase was illustrated and described as ‘prominent’. Unlike our current study, the study by Cabezas et al. did not use a defined scoring system for assessing the intensity of reaction, so it is not clear what level of immunostaining was considered prominent. From a pathological point of view, the whole point of staining a sample is to bring the presence of the marker (in this case SOX2) to prominence. After a detailed study of Cabeza's figure showing SOX2 expression, we believe that the demonstrated positivity corresponds to weak (or moderate at most) staining. It would be therefore interesting to determine whether the aforementioned weak staining detected in the early luteal phase represents true positivity or faint non‐specific staining that is typically seen in various cellular compartments of different cell types (epithelial as well as mesenchymal). In this and other aspects (discussed further below), the comparison of previous results with those of our current study is limited. In both our current study and that performed by Cabezas et al., SOX2 glandular positivity was localized to different cellular compartments. While Cabezas et al. demonstrated nuclear expression using a mouse monoclonal antibody, the SOX2 antibody employed in our study (polyclonal with 100% predicted reactivity for pigs and cows) showed cytoplasmic staining. Most notably, the report by Cabezas et al. lacks information about the topography of SOX2 expression in the bovine endometrium. The authors described positivity in glandular and some stromal cells, without providing details specifying whether entire glands or only some parts of them were positive. In our current study, we found marked differences in SOX2 expression between individual portions of the endometrial glands. While the basal portions showed strong diffuse positivity, the number of SOX2‐positive cells and the intensity of immunoreaction were lower in the luminal two thirds of the glandular epithelium

A second study reporting SOX2 expression in bovine endometrium was published by Lara et al. in 2016 (Lara et al., 2017). Lara et al.’s study included animals in the follicular phase of the oestrous cycle. The authors reported SOX2 positivity in both glandular and some stromal cells. In the endometrial glandular cells, positivity was detected in the nuclear and perinuclear areas; however, other descriptive parameters of the immunoreaction are lacking in the study (including the intensity of reaction, percentage of SOX2‐positive cells and topography of SOX2 expression). Lara et al. also described SOX2 positivity in the ‘glandular lumen transmembrane area’, but the exact area this statement applies to is unclear. Immunohistochemical analysis traditionally distinguishes between nuclear, cytoplasmic and membranous positivity. Examining the figures in Lara et al.’s study, it is clear that the glandular cells showed only weak cytoplasmic positivity, while the nuclei and cytoplasmic membranes were SOX2 negative. However, one fact caught our attention. Both research groups (Cabezas et al. and Lara et al.) used an identical SOX2 antibody (clone, manufacturer and dilution), so it is not clear why expression in the glandular epithelium was detected in different cellular compartments in each of these studies (nuclear positivity in Cabezas et al.’s study and cytoplasmic positivity in Lara et al.’s study). Given the compelling nuclear positivity demonstrated in Cabezas et al.’s article, we believe that this particular antibody only specifically detects SOX2 in the nucleus. Therefore, we consider weak cytoplasmic positivity to be non‐specific, which is supported by the large number of positive stromal cells in the surrounding tissue reported in Lara et al.’s study.

In the third study by Lupicka et al., there were insufficient data on SOX2 immunoexpression in bovine uterus (Łupicka et al., 2015). The authors described staining mainly in myometrium and illustrated SOX2 positivity in leiomyocytes only. A more detailed description of SOX2 immunohistochemistry in the endometrium is missing from the study. For this reason, it is not possible to compare the results of Lupicka et al.’s study with those of our current study. Overall, we conclude that our study is only the second to reliably demonstrate SOX2 immunopositivity in bovine endometrial tissue. Finally, none of the above‐mentioned studies investigated SOX2 expression in both the follicular and luteal phases of the oestrous cycle.

To the best of our knowledge, our present study is the first to demonstrate SOX2 expression in the glandular component of the porcine endometrium. Interestingly, all cases employed showed an almost identical expression pattern to that of SOX2 in bovine endometrial samples (i.e., strong diffuse expression found only in the basal portions of the endometrial glands). For endometrial stromal cells, we found random and sporadic SOX2 expression without apparent clustering of positive cells. This is the first time endometrial stromal cells have been reported to be immunohistochemically positive for SOX2 expression, although its expression in pigs was previously demonstrated by western blotting and polymerase chain reaction (Subbarao et al., 2015). The detection of SOX2 in both the endometrial stroma and glands suggests that, as in humans, two different stem cell populations, namely epithelial and mesenchymal, may be present in the endometrium of pigs and cows. The fact that all cases of both animal species employed showed identical SOX2 expression patterns indicates its consistent expression throughout the oestrous cycle (without differences between the follicular and luteal phases of the cycle). The SOX‐2 immunohistochemistry performed in our study does not determine the percentage of epithelial stem cell population in porcine and bovine endometrium. In humans and pigs, no differences in clonogenicity of either endometrial epithelial or stromal cells have been reported between the follicular and secretory phases of the menstrual and oestrous cycles (Masuda et al., 2010; Schwab et al., 2005).

The basalis glandular epithelium is the postulated site of endometrial epithelial progenitor cells in humans (Garget). Unfortunately, no progress has yet been made in identifying a specific marker of this glandular cell population in humans or animals. Based on the results of our current study, we believe that SOX2 could be a promising marker for identifying basal portions of endometrial glands in pigs and cows.

It is speculated that undifferentiated endometrial stem cells are less sensitive to sex hormones than their terminally differentiated daughter cells due to a lack of hormone receptor expression (Garget et al., 2008). Regarding the immunohistochemical analysis of hormone receptors in bovine and porcine endometrium, the following results were obtained in our current study. Staining with PR revealed an inverse expression pattern to that of SOX2 in both the bovine and porcine endometrium. Specifically, strong PR expression was found in the middle and luminal portions of the endometrial glands, while the intensity of the reaction and the percentage of marker‐positive cells were lower in the basal portions. ER staining revealed several expression patterns, one of which resembled that of the PR. However, the differences between ER expression in the individual portions of the glands were not as obvious as those observed for the PR. To confirm that the ER and PR staining in the basal portions of the glands did not decrease artificially, we compared staining in the glandular epithelium of each sample with that in the myometrium. The moderate to strong diffuse nuclear labelling in the myometrium served as a positive internal control. Loss of hormone receptor expression in leiomyocytes would indicate false negative results. However, we found an abrupt transition between the weakly stained basally located glandular epithelium and strongly stained neighbouring leiomyocytes, which supports the accuracy of our results.

The comparison of SOX2 and hormone receptor expression between porcine and bovine endometrium was as follows. For SOX2, our study did not reveal differences between pigs and cows. An identical expression pattern and virtually the same number of SOX2‐positive cells were found in different portions of the endometrial glands in both animal species. In contrast, minor differences were found for progesterone receptors. While the expression pattern was the same in both the porcine and bovine endometrium (i.e., strong and extensive PR expression limited to the middle and luminal portions of the endometrial glands), the basal glandular portions in cows showed significantly lower number of PR‐positive cells compared to pigs. ER immunohistochemistry revealed similarity between porcine and bovine endometrium in one expression pattern in which pigs were found to have a slightly lower number of ER‐positive cells in basal glandular portions compared to cows. The other two porcine ER expression patterns differed from the remaining bovine pattern mainly by the strong intensity of immunoreaction. Thus, our study points to minor differences in hormone receptor status between porcine and bovine endometrium.

In general, the differences in ER expression may be due to technical problems or functional reasons. Given that ER immunohistochemistry was performed under identical methodological procedures, we believe that the differences between the individual ER expression patterns found in our current study were due to functional reasons. Regarding the bovine endometrium, patterns 1 and 3 were very similar, while in the second pattern, the most striking difference was the decrease in the percentage of ER‐positive cells and the intensity of the immunoreaction in the basal endometrial portions. In pigs, the differences between the two expression patterns were relatively discrete. Minor changes in ER expression may reflect slight interindividual differences in hormone receptor status in bovine and porcine endometrium. The age of the animals could theoretically be another reason for the differences between ER expression patterns. There are currently no data comparing the number of endometrial epithelial stem cells in animals of different ages. Thus, the question is whether the proportion of endometrial stem/progenitor cell population, which is characterized by a lesser amount of receptor content, is age‐dependent. In our study, the second ER expression pattern, which was similar to that of the PR, was found mainly in metestrus and dioestrus (corresponding to the luteal phase of the oestrous cycle). In one recent study, the authors reported different hormonal expression patterns in eutopic and ectopic endometrium in humans during the menstrual cycle (Lenz et al., 2021). As in our current study, a significant decrease of ER expression was found in the secretory phase of the menstrual cycle.

Inverse correlation of SOX‐2 and hormone (especially progesterone) receptors in both the porcine and bovine endometrium found in our current study could be related to the *FOXA1* gene. This gene, also known as hepatocyte nuclear factor 3α, is involved in regulating the embryogenesis of various tissues as well as playing an important role in the post‐natal development of hormone‐dependent tissues such as prostate or mammary gland (Costa et al., 1989). Recently, attention has been focused on investigating the role of FOX1A gene in the pathogenesis of certain cancer types. In breast cancer, a positive correlation between FOXA1 and ER expression has been reported (Badve et al., 2007). Regarding the association between SOX2 and FOX1A, one recent study found a negative regulation of FOX1A by SOX‐2 in human breast and lung cancer (Li et al., 2014). Thus, the question is whether the inverse correlation of SOX‐2 and hormone receptors in bovine and porcine endometrium is functionally linked to the *FOXA1* gene.

Overall, our study demonstrates an inverse correlation between the expression patterns of SOX2 and hormone receptor expression in both bovine and porcine endometrium. SOX2‐positive glandular cells in the basal portions of the endometrium expressed lower levels of hormone receptors (especially PR) than in the middle and luminal portions of the endometrial mucosa. Down‐regulation of hormone receptors has been used to indicate a less differentiated cell phenotype. Therefore, our results support the existence of epithelial stem/progenitor cells in the porcine and bovine endometrium, and also suggest their possible localization in the basal portion of the endometrial mucosa. These findings in farm animals are surprising for two reasons. First, the endometrium of pigs and cows is not structurally and functionally divided into the basalis and functionalis, and second, the endometrial tissue is resorbed (not shed) during the oestrous cycle. By contrast, putative epithelial stem cells in the human endometrium are thought to reside in the basalis, allowing the glandular epithelium of the functional layer to be replenished and regenerated during the proliferative phase of the menstrual cycle. This hypothesis is also supported by some immunohistochemical investigations (Fayazi et al., 2016). Thus, from the endometrial epithelial stem cell perspective, the data obtained in our study point to a similarity between the human endometrium and that of pigs and cows.

## 5 CONCLUSION

Our data support the presence of two stem cell populations in porcine and bovine endometrium, one of epithelial origin and one of mesenchymal (stromal) origin. As far as we know, our current study is the most thorough investigation of the porcine and bovine endometrium, focusing on endometrial epithelial stem/progenitor cells, using an immunohistochemical assay. The inverse expression patterns of hormone (especially progesterone) receptors and the embryonal stem cell marker SOX2 suggest that endometrial epithelial stem/progenitor cells represent a subset of epithelial cells that reside in the basal portions of the endometrial glands. SOX2 appears to be a promising marker for identifying the basal portions of the endometrial glands. The present study is the first to address the epithelial stem/progenitor cell population in the porcine endometrium. Further research focusing on protein/gene expression of selected stem cell markers in the bovine and porcine endometrium is required, with a focus on the epithelial stem cell subpopulation.

## CONFLICT OF INTEREST

The authors declare no conflict of interest.

## AUTHOR CONTRIBUTIONS

*Project development, manuscript writing and editing*: Jiri Lenz. *Manuscript writing, literature (PubMed) search, tables and graphs*: Petra Konecna. *Manuscript editing, critically revising the article*: Frantisek Tichy. *Manuscript writing, histological images, literature (PubMed) search*: Dominka Machacova. *Statistical analysis and manuscript writing*: Ludek Fiala. *Histological images and statistical analysis: Pavel Hurnik*. *Manuscript editing and critically revising the article*: Michal Kyllar.

## ETHICS STATEMENT

All the experimental procedures including tissue sampling were conducted according to the ARRIVE guidelines, U.K. Animals (Scientific Procedures) Act, 1986 and the associated guidelines, EU Directive 2010/63/EU for animal experiments and Guide for the Care and Use of Laboratory Animals. The study was approved by the Ethics Committee of the Faculty of Veterinary Medicine, University of Veterinary Sciences Brno.

### PEER REVIEW

The peer review history for this article is available at https://publons.com/publon/10.1002/vms3.802.

## Data Availability

The datasets generated during and/or analyzed during the current study are available from the corresponding author on reasonable request.
